# Fatigue in Multiple Sclerosis: A Review of the Exploratory and Therapeutic Potential of Non-Invasive Brain Stimulation

**DOI:** 10.3389/fneur.2022.813965

**Published:** 2022-04-28

**Authors:** Samar S. Ayache, Nicolas Serratrice, Georges N. Abi Lahoud, Moussa A. Chalah

**Affiliations:** ^1^EA4391 Excitabilité Nerveuse and Thérapeutique, Université Paris Est Créteil, Créteil, France; ^2^Department of Clinical Neurophysiology, DMU FIxIT, Henri Mondor University Hospital, Assistance Publique-Hôpitaux de Paris (APHP), Créteil, France; ^3^Department of Spine Surgery, Centre Médico Chirurgical Bizet, Paris, France; ^4^Institut de la Colonne Vertébrale et des Neurosciences (ICVNS), Centre Médico Chirurgical Bizet, Paris, France

**Keywords:** multiple sclerosis, fatigue, neuromodulation, corticospinal excitability, tDCS, TMS, tRNS

## Abstract

Fatigue is the most commonly reported symptom in patients with multiple sclerosis (MS). It is a worrisome, frequent, and debilitating manifestation that could occur at any time during the course of MS and in all its subtypes. It could engender professional, familial, and socioeconomic consequences and could severely compromise the patients' quality of life. Clinically, the symptom exhibits motor, cognitive, and psychosocial facets. It is also important to differentiate between perceived or subjective self-reported fatigue and fatigability which is an objective measure of decrement in the performance of cognitive or motor tasks. The pathophysiology of MS fatigue is complex, and its management remains a challenge, despite the existing body of literature on this matter. Hence, unraveling its neural mechanisms and developing treatment options that target the latter might constitute a promising field to explore. A PubMed/Medline/Scopus search was conducted to perform this review which aims (a) to reappraise the available electrophysiological studies that explored fatigue in patients with MS with a particular focus on corticospinal excitability measures obtained using transcranial magnetic stimulation and (b) to assess the potential utility of employing neuromodulation (i.e., non-invasive brain stimulation techniques) in this context. A special focus will be put on the role of transcranial direct current stimulation and transcranial magnetic stimulation. We have provided some suggestions that will help overcome the current limitations in upcoming research.

## Introduction

Multiple sclerosis (MS) is one of the most common neurological diseases and a serious cause of disability in young adults. Its natural course is characterized by recurrent relapses and progressive functional decline (1). With disease evolution, patients could accumulate several neurological dysfunctions, including motor deficit, sensory dysfunction, and sphincter disorders, among others (2). In addition, they could suffer from several “silent” “non-motor” complications, such as fatigue, pain, emotional manifestations, and cognitive dysfunctions (3).

Over the last two decades, MS symptoms have preoccupied the scientific community, and tremendous efforts have been made to understand the reasons behind their development and the modalities of their treatments. Among these symptoms, fatigue constitutes a real enigma and has given rise to collective awareness. Although the last few years have shown a growing literature on the characterization, pathophysiology, and treatment of MS fatigue, this symptom continues to challenge the medical and research societies of its difficult-to-treat nature and its resistance to the available pharmacological solutions. Hence, in this review, we will start with a definition of MS fatigue by highlighting the difference between fatigue and fatiguability. Then, we will give an overview of its underlying pathophysiological mechanisms. There will be particular focus on the application of the neurophysiological techniques in this domain. Afterward, we will address the place of non-invasive brain stimulation (NIBS) interventions in the treatment of this symptom.

## Fatigue in MS

MS fatigue is very common; it could impact the lives of 75–90% of patients suffering from this disease (2, 4). It deeply affects their professional, social, and familial domains and could result in significant health costs and, therefore, should not be neglected (5, 6). For all these reasons, understanding this symptom and adopting novel therapeutic approaches have become more important than ever before.

To start, the definition of fatigue has been a source of confusion for several years. On the one hand, the terms “tiredness,” “malaise,” and “motor weakness” have been interchangeably used by patients to describe their fatigue; on the other hand, care providers have sometimes perceived fatigue as a lack of self-motivation. Toward the end of the 90's, a consensus was set by the MS Council for Clinical Practice Guidelines and has ended this debate (7). According to this council, MS fatigue corresponds to “a subjective lack of physical and/or mental energy that is perceived by the individual or caregiver to interfere with usual and desired activities.” Currently, it is recommended to adopt this definition as has been thoroughly discussed in Mills and Young's study (8). In the same perspective of this definition, the intensity of MS fatigue is temperature-dependent in a way that hot or cold temperatures would worsen or alleviate fatigue, respectively. This aspect differentiates it from the “classical” tiredness encountered in healthy individuals.

In addition to the importance of setting a clear definition of fatigue, it is important to stress the difference between subjective or perceived self-reported fatigue and fatigability. While the former reflects a subjective experience that is classically tested by self-administered questionnaires, the latter reflects a performance decrement during the execution of a task and is usually evaluated with various cognitive or physical exercises.

Fatigue is a multifaceted symptom and consists of three domains: the physical, psychosocial, and cognitive domains. Thus, when patients complain about fatigue, the clinician or researcher should understand whether they feel this fatigue in the three domains or whether it only concerns one domain, for instance, the cognitive one. For this reason, some of the self-rated questionnaires that have been developed to diagnose and follow up on this complaint included questions dedicated to the assessment of several aspects of MS fatigue. For instance, the Modified Fatigue Impact Scale (MFIS), one of the most widely used scales, includes 21 questions that examine the three facets of fatigue (i.e., the physical, psychosocial, and cognitive ones) (7). In a similar manner, the Fatigue Scale for Motor and Cognitive Functions includes 20 questions and assesses two dimensions of MS fatigue as its name implies (9). Other scales assess one dimension of fatigue (e.g., the physical dimension), such as the 9-item Fatigue Severity Scale, which is one of the first tools developed to be used in PwMS (10), while others such as the Visual Analog Scale [VAS, (11)] provide a global assessment of this symptom [For a review refer to (1)].

Moreover, when talking about MS fatigue, it is pertinent to distinguish between primary fatigue, which is related to disease-specific mechanisms, and secondary fatigue, which could rather be attributed to comorbidities (motor symptoms, psychiatric manifestations, other medical conditions, or treatments adverse events) (1).

## Selection Criteria

Research was done following PRISMA guidelines using computerized databases (PubMed/MEDLINE, Scopus) (12). An independent review was conducted by two of the authors (SSA and MAC) in order to identify original research articles published in English and French languages at any time till November 2021. The following key terms were used: (“MS” OR “multiple sclerosis”) AND (“fatigue”) AND (“non-invasive brain stimulation” OR “NIBS” OR “transcranial magnetic stimulation” OR “TMS” OR “theta burst stimulation” OR “TBS” OR “motor evoked potential” OR “MEP” OR “cortical excitability” OR “corticospinal excitability” OR “intracortical inhibition” OR “intracortical facilitation” OR “silent period” OR “interhemispheric inhibition” OR “transcranial direct current stimulation” OR “tDCS” OR “transcranial random noise stimulation” OR “tRNS”). In order to look for additional sources, the bibliographical references of the retrieved articles were also scanned.

## Pathophysiology of Fatigue in MS

Clinical, neuropsychological, neuroanatomical, neuroimmune, and neurophysiological studies attempted to explore this multidimensional symptom. From a clinical perspective, the relationship between fatigue and physical disability appears to be inconsistent; MS fatigue seems to occur in all disease subtypes (4). From a neuropsychological viewpoint, fatigue could be associated with specific emotions, thoughts, and behaviors according to a cognitive-behavioral model proposed by van Kessel and Moss-Morris (13). In addition, this symptom could be associated with emotional factors, with which it may have bidirectional relationships and may share common biological substrates (14). In terms of neuroanatomy, inconsistencies exist regarding conventional measures (e.g., lesion load, global brain atrophy), but more advanced neuroimaging modalities (e.g., tractography, normal-appearing white matter, regional brain volumes and lesion load, brain activity, and functional connectivity at rest or during task performance) have unraveled a cortico-striato-thalamo-cortical loop related to MS fatigue (15–19). The exploration of neuroimmune and neuroendocrine axes has yielded scarce findings linking MS fatigue to some peripheral proinflammatory cytokines (20–22), while the relationship between this symptom and other outcomes were inconsistent {i.e., cerebrospinal fluid markers, orexin-A system, hypothalamus-pituitary-adrenal axis (20, 21, 23, 24), or absent [peripheral T cell populations or markers of inflammation (25, 26)]} [for reviews see (14)]. Finally, neurophysiology also constitutes a discipline that addresses MS fatigue in terms of pathophysiology and management as will be developed in the following sections.

## Nibs to Explore and Manage Fatigue in MS

Modulating the activity of brain regions and circuits continues to be a fascinating scientific field and a source of inspiration for researchers worldwide. The story began in the previous century when scientists first tested the impact of a weak electric current on the functioning of neural networks in animals and discovered that the application of a polarizing current on the scalp results in various effects on cortical activity. Afterward, much research has taken place across the world, and the fruit of this long investment has resulted in the development of the various NIBS techniques that we currently have at our disposal. Among these techniques, two are particularly interesting and have been the subject of many scientific investigations into different pathologies. The first is based on a famous law of biophysics—Faraday's law (the law of electromagnetic induction)—, while the second rather uses a weak electric current. These are, respectively, the transcranial magnetic stimulation (TMS) and the transcranial direct current stimulation (tDCS) techniques (27–30).

### Neurophysiology of Fatigue in MS Using NIBS

As stated previously, TMS finds its roots in an ancient law of biophysics—the Faraday law. In fact, this law paved the way for the development of what has now become the rescue solution to some crippling neuropsychiatric manifestations, such as depression and neuropathic pain (29). Briefly, Faraday demonstrated that making an electric current flow in a conductive element would induce a magnetic field; the latter could in its turn induce an electric field in another conductive element placed nearby. Hence, applying a magnetic field on the scalp would diffuse toward the underlying cortical networks and would stimulate the corresponding nervous fibers (29).

The first clinical development of TMS served for the study of pyramidal motor conductions, using the technique known as motor evoked potentials (MEP). Performing MEP remains the most common application of TMS (31). This technique uses unique shocks applied to the skull to stimulate the pyramidal cortical neurons and to the spine to activate the nerve roots. A surface electromyographic recording is made at the level of the muscles of interest and the parameters (i.e., latency and amplitude) of the evoked responses are generally measured. Central motor conduction time (CMCT) is another TMS parameter used in clinical settings and reflects the time the nerve impulses take to travel from the motor cortex to the spinal motor neurons. It could be measured by subtracting the MEP latency obtained from spinal magnetic stimulation (also known as peripheral motor conduction time) from TMS-evoked MEP latency (32). Its lengthening may arise from a degeneration or a demyelination affecting the fastest-conducting cortico-motoneuronal fibers (33). Prolonged MEP latency was found to be a significant predictor of fatigue severity (34) while CMCT seems to be unrelated to this symptom (35).

Apart from obtaining conventional MEP, TMS has other important applications such as studying cortical excitability, which assesses different processes of regulation and execution of motor commands using paradigms of single and double cortical pulses. The parameters measured (i.e., motor thresholds (MT), short-interval intracortical inhibition (SICI) and intracortical facilitation (ICF), cortical silent period (CSP), interhemispheric inhibition (IHI), cerebello-cortical inhibition, among others) provide information on neuronal modulation circuits using well-characterized GABAergic, glutamatergic, or cholinergic neurotransmission (32, 33). Excitability paradigms have been widely used to examine the pathophysiological processes behind several neurological and psychiatric symptoms, among which stands MS fatigue.

To start, concerning single-pulse parameters, no correlation was found between fatigue severity and resting MT (rMT) (36, 37), a parameter that reflects the excitability of the corticomotor neuronal membrane, including the spinal level. It corresponds to the stimulation intensity, as a percentage of the maximal stimulator output, that yields MEP of at least 50 μV amplitude on a fully relaxed muscle in 5 out of 10 trials (32, 33). The CSP is another single-pulse parameter that reflects cortical (GABA-B) and spinal inhibitions (e.g., Renshaw cells, IA inhibitory interneurons) (32, 33). It could last up to 300 ms and corresponds to the interruption of voluntary muscular activity in a muscle of interest by applying a TMS pulse over the contralateral motor cortex. Prolonged CSP was found to be associated with fatigue severity in one study (38) but not in another one (37). Such discrepancy could be related to the clinical and methodological differences between both studies, such as the cohorts' disease characteristics (predominantly relapsing–remitting vs. progressive disease, respectively) and the adapted fatigue measures (VAS vs. MFIS, respectively).

Besides single-pulse measures, double-pulse measures could also be used. Some of them consist of applying a first subthreshold conditioning stimulus (whose intensity is below the rMT) that would inhibit or facilitate the response of a second suprathreshold stimulus (whose intensity is above the rMT) delivered to the same cortical site depending on the interstimulus interval (ISI) (32). For instance, applying short (≤ 6 ms) and long (≥7 ms) ISI could yield SICI and ICF which respectively, reflects GABA-A and glutamatergic transmissions. IHI represents another double-pulse measure that consists of conditioning the response of a suprathreshold stimulus by applying a suprathreshold stimulus over the contralateral motor site, and reflects GABAergic transcallosal activity (32). Three works have explored SICI in the context of MS fatigue and found low (36), high (37), or similar (39) pattern of inhibition in fatigued PwMS compared to non-fatigued PwMS and/or healthy controls (HC). In addition, in these three works, no significant group difference (or correlation) was obtained in terms of fatigue and ICF. Moreover, one of the three works included an IHI assessment and found no correlation between this measure and fatigue severity (37).

Some studies also explored the neurophysiological correlates of MS fatigue during motor task performance and tested the relationship of this symptom with movement preparation or execution phases. Some reported positive findings linking MS fatigue to movement-related TMS outcomes. Premovement MEP facilitation which is a normal finding following a motor task was found to be significantly reduced in fatigued PwMS compared to their non-fatigued counterparts and HC (39–41). This finding was correlated with frontal lesion load (39), motor performance decay [decrease in movement rate, (40)], and fatigue severity (39), suggesting a relationship between MS fatigue and abnormalities involving cerebral networks devoted to movement preparation. In addition, higher MEP amplitude was observed with contralateral hand grip following the fatiguing task in HC and non-fatigued PwMS but not in fatigued PwMS. This finding might suggest an involvement of callosal dysfunction in MS fatigue (42). Moreover, fatigue severity seems to be correlated with the time required for the rMT to reach the pre-exercise level (36). Conversely, no group difference in post-exercise MEP facilitation was found between non-fatigued and fatigued PwMS (43).

The second other major application of TMS is the realization of repetitive TMS (rTMS). Briefly, this method consists of delivering trains of stimulation at various frequencies and requiring specific machines (27, 29). Data on rTMS effects derive from numerous studies performed in healthy individuals, in whom low and high frequency (LF and HF) rTMS, respectively led to reduction and augmentation of MEP size. Hence, LF-rTMS and HF-rTMS have been perceived as inhibitory and facilitatory interventions. However, this viewpoint is simplistic, and it is now known that this dichotomy is no more valid since rTMS effects also depend on the baseline excitatory state of the nervous circuits; a state that would vary between individuals and even in the same subject at different moments of the day, it would also vary between healthy networks and those affected by various pathologies. Even more, several studies have documented that the augmentation/reduction of MEP amplitude after the application of HF/LF rTMS over the precentral cortex [i.e., the primary motor cortex (M1)] may be due to a decrease/increase of the GABA mediated inhibitory control of the corticospinal circuit rather than a direct modulation of the motor cortex excitability. Thus, what is perceived as “facilitatory” protocol (HF rTMS) could be in fact “inhibitory” (decrease in the functioning of the GABA interneurons) and vice versa. Other factors that can impact rTMS effects include age, drugs, and genetic factors, among others [For review, please refer to (27)].

In addition to the “classical” rTMS paradigms, a particular form of rTMS has been recently developed, the so-called theta burst stimulation (TBS). It consists of applying bursts (three pulses per burst at 50 Hz) in a repetitive manner at theta frequency (at 5 Hz) (44). TBS could induce changes in corticospinal excitability and the nature of such changes depends on the way the bursts are applied. Continuous and intermittent TBS (cTBS and iTBS) could lead to long-term depression-like and long-term potentiation-like effects, respectively (44).

Concerning rTMS and MS fatigue pathophysiology, it is worth noting here that some study protocols have tested the effects of 5-Hz rTMS over MEP outcomes in PwMS. In one study, MEP outcomes did not significantly differ between fatigued and non-fatigued PwMS, with both patient groups showing an increase in MEP amplitude following the intervention (39). In another study, the expected increase in MEP size was not obtained in fatigued PwMS, an increment that was found in their non-fatigued counterparts and in HC (45). Methodological differences could partly account for the observed changes as the second study included an attentional task (instructions to focus attention on the hand corresponding to stimulation); in addition, as aforementioned, inter-individual variability in terms of the baseline cortical excitability level could be behind such a discrepancy. One should note that the second study also assessed the impact of paired-associative stimulation (peripheral nerve stimulation followed by 5-Hz rTMS) on MEP amplitude and yielded similar findings (i.e., no change in MEP amplitude in fatigued PwMS) (45).

As for TBS, it is worth mentioning that no single study has applied this technique to explore the underlying mechanisms of MS fatigue. Its future application in this context could unveil additional mechanisms incriminated in the generation of this symptom. Table 1 summarizes the neurophysiological studies that explored MS fatigue.

**Table 1:** Summary of studies on neurophysiological parameters in MS fatigue.

	**Participants**	**Neurophysiological parameters**	**Other parameters**	**Results**
Colombo et al. (46)	30 PwMS (15 non-fatigued and 15 fatigued, FSS) Immunomodulant/immunosuppressive drugs: None	MEP of the four limbs	Disability: EDSS Function: Pyramidal functional system score Depression: MADRS	Higher MEP abnormalities in fatigued (*n* = 5) vs. non-fatigued (*n* = 1) PwMS Higher lesion loads (parietal lobe, internal capsule, periventricular trigone) in fatigued vs. non-fatigued PwMS Significant association between fatigue scores and MRI lesions burden
Petajan and White (42)	32 PwMS (Classified according to presence or absence of upper extremities weakness) Immunomodulant/immunosuppressive drugs: not provided 10 HC	Evaluation before and after fatiguing exercise of resting and facilitated MEP using TMS (abductor pollicus brevis and flexor carpi radialis)	Exercise-induced changes in energy metabolism (phosphocreatine) measured using 31P magnetic resonance spectroscopy in flexor carpi radialis	Lower peak force, faster decline in force, and prolonged CMCT in PwMS vs. HC Higher MEP amplitude was observed with contralateral hand grip following the fatiguing task in HC and non-fatigued PwMS but not in fatigued PwMS. No group differences in phosphocreatine outcomes.
Romani et al. (47)	60 PwMS (20 fatigued and 40 fatigued, fatigued having FSS scores above the 75^th^ of a previous sample) Immunomodulant/immunosuppressive drugs: None	Evaluation before and after 8-week treatment with 4-aminopyridine and fluoxetine (no placebo control): Somatosensory evoked potentials, TMS, muscle fatigability	Other fatigue measures: FIS Depression: HDRS, BDI Disability: EDSS	At baseline, fatigue test scores consistently correlated with depression and cognitive test scores but not with the fatigability test. Fatigue scores decreased in both treatment groups in a similar way. Due to the design of the study, this cannot be disjoined from a placebo effect. The changes in fatigue scores could not be predicted in the FLX group, whereas in the 4-AP group, higher basal fatigability test scores were associated with a greater reduction in fatigue scores
Perretti et al. (43)	41 PwMS (9 non-fatigued and 32 fatigued Immunomodulant/immunosuppressive drugs: all receiving interferon beta-1a 13 HC	MEP at rest, post-exercise MEP facilitation (PEF), and post-exercise MEP depression (PED)		Reduction in MEP (depression) following fatigue onset in HC but not in PwMS No group difference in post-exercise MEP findings (facilitation) between non-fatigued and fatigued PwMS
Liepert et al. (36)	16 PwMS (8 fatigued and 8 non-fatigued based on FSS (fatigued had FSS ≥4) Immunomodulant/immunosuppressive drugs: not provided 6 HC	rMT SICI ICF Three-time measurements in relation to an exercise (repeating hand grip): pre-exercise, postexercise (when rMT was back to the postexercise level), and 15 min later CMAP of the abductor pollicis brevis (following median nerve stimulation) Before and after exercise		At baseline: SICI was lower in fatigued PwMS compared to the other groups After exercise: SICI remained lower in the fatigued group in comparison with their non-fatigued counterparts and HC Fatigue severity correlated with the time required for the rMT to reach the pre-exercise level
Santarnecci et al. (48)	10 PwMS (fatigue measured using FSS and FIS but not classified according to fatigue scores was performed) Immunomodulant/immunosuppressive drugs: 5 patients receiving β-interferon 10 HC	CSP recorded at the first dorsal interosseus muscle and at the abductor digiti minimi at baseline and after a fatiguing tapping task CSP and fatigue changes before and after chronic amantadine therapy in PwMS	Sleep: ESS Anxiety: Hamilton scale for anxiety Depression: HDRS and BDI	Prior to amantadine therapy: shorter CSP in PwMS vs. HC at baseline and contrasting pattern of CSP changes following fatiguing task in PwMS (increase) and in HC (decrease) After amantadine therapy: • Significant improvement in FSS and marginal improvement in FIS •Normalization of CSP duration in PwMS • Correlation between CSP changes and fatigue improvement (only with FIS, only in the first dorsal interosseus muscle).
Morgante et al. (39)	33 PwMS (17 non-fatigued and 16 fatigued, fatigued had FSS>4) Immunomodulant/immunosuppressive drugs: 32 patients receiving treatments 12 HC	MRI: lesion load TMS: CMCT, SICI, ICF, pre-movement facilitation, and effect of short trains of 5-Hz repetitive TMS	Depression: HDRS	No significant group differences in depression scores Higher frontal lobe LL in fatigued PwMS No significant group difference in SICI/ICF Absence of MEP size increase following repetitive TMS in PwMS compared to HC Lack of pre-movement facilitation in fatigued PwMS vs. non-fatigued PwMS and HC Correlation between pre-movement facilitation abnormalities, frontal LL, and fatigue severity
Scheidegger et al. (49)	23 PwMS Immunomodulant/immunosuppressive drugs: 14 patients receiving the treatment 13 HC	TMS: CMCT by means of the triple stimulation protocol and obtaining central conduction index (CCI) during a fatiguing exercise of the abductor digiti minimi (2 min) followed by recovery (7 min)		No significant group difference in force decline following exercise Less marked CCI decline in PwMS compared to HC No correlation between fatigue scores and CCI or force drop
Russo et al. (40)	24 PwMS (12 non- fatigued and 12 fatigued; fatigued had FSS > 36) Immunomodulant/immunosuppressive drugs: information not provided 10 HC	Motor cortex excitability and the premovement facilitation (PMF) before and after the finger-tapping task		Reduction of correct sequences and the ability to keep a fixed movement rate in fatigued vs. non-fatigued PwMS as well as HC Reduction of post-exercise PMF among fatigued PwMS Correlation between PMF abnormalities and performance decay
Thickbroom et al. (50)	10 PwMS Immunomodulant/immunosuppressive drugs: 9 patients receiving β-interferon 13 HC	MEP amplitudes before and after each cycle of a foot-tapping task	Five cycles of 15 s-foot tapping task followed by 45 min rest period: maximum voluntary contraction of ankle dorsiflexion (at baseline and immediately after the completion of the task) Number of taps Inter tap interval	Increase in MEP amplitudes following exercise in both groups, but more important in PwMS Maximal voluntary contraction is lower in PwMS vs. HC. Decreased maximal voluntary contraction after exercise in both groups but more important in PwMS. No difference was found in the tapping rate.
Conte et al. (45)	25 PwMS (13 non-fatigued and 12 fatigued based on MFIS (i.e., details NP) Immunomodulant/immunosuppressive drugs: patients receiving treatment (without further information) 18 HC	Experimental conditions (relaxed vs. attention): 5-Hz repetitive TMS and paired associative stimulation while focusing attention on the hand contralateral to the stimulated motor cortex		Absence of attention-induced MEP increase using both techniques in fatigued PwMS compared to non-fatigued patients Correlation between attention-induced repetitive-TMS related changes and fatigue severity (mostly physical subscale)
Russo et al. (41)	30 PwMS (non-fatigued and fatigued based on FSS (i.e., fatigued patients had FSS ≥4) Immunomodulant/immunosuppressive drugs: information not provided	Pre movement facilitation	DTI study	Significant difference in premovement facilitation between fatigued and non-fatigued groups Significant correlation between fatigue scores and mean diffusivity in bilateral fronto-thalamic connections
Chaves et al. (51)	82 PwMS Immunomodulant/immunosuppressive drugs: 47 patients receiving treatment	Bilateral aMT and rMT Then ratios were calculated between weaker and stronger side aMT and rMT (Weaker and stronger sides were defined according to performance on pinch and hand grip)	Disability: EDSS Dexterity: 9HPT Cognition: SDMT Walking speed: instrumented walkway Heat sensitivity: VAS Fatigue: VAS Pain: VAS Subjective impact of MS: MSIS-29	Higher excitability in the hemisphere controlling the weaker side Shifting of this asymmetry (i.e., lower excitability in the hemisphere responsible for the weaker side) predicted more severe MS related symptoms and may hint toward a neurodegenerative process
Chaves et al. (38)	82 PwMS Immunomodulant/immunosuppressive drugs: 48 patients receiving treatment	Bilateral aMT, rMT and CSP	Fatigue: VAS Exercise test inflammatory cytokines: TNF	Poor fitness was found in the majority of patients. A link seems to exist between fitness level and CSP (i.e., low level predicted longer CSP) and between CSP and fatigue.
Mordillo-Mateos et al. (35)	17 PwMS Immunomodulant/immunosuppressive drugs: 11 patients receiving treatment 16 HC	CMAP and F wave of right and left first dorsal interosseous muscles (after ulnar nerve stimulation) rMT, MEP amplitude latency (at 120% rMT) and CMCT of above-mentioned muscles These parameters were measured before, immediately, one and two minutes after the fatiguing task	Fatigue: FSS Fatigue: BRPES Motor performance: maximal hand grip, and motor decay	At baseline: lower CMAP and MEP, higher RMT, longer CMCT and higher fatigue scores in PwMS compared to HC Task performance: lesser handgrip strength in PwMS compared to HC In PwMS, fatigue shown to be independent from handgrip strength; fatigue shown to be independent from CMCT
Chalah et al. (37)	38 PwMS [17 non-fatigued and 21 fatigued based on MFIS (i.e., Fatigued: MFIS ≥ 45)] Immunomodulant/immunosuppressive drugs: 19 patients receiving treatment	rMT CSP SICI ICF IHI	Neuropsychological parameters: Anxiety and Depression: HADS Excessive Daytime sleepiness: ESS Cognition: SDMT Alexithymia: TAS Neuroradiological measures (Volume based morphometry)	Higher SICI in fatigued patients compared to their non-fatigued counterparts. Fatigued patients also showed higher HADS and TAS scores, as well as larger caudate nuclei and smaller left parietal cortex.
Coates et al. (34)	26 PwMS (13 non-fatigued and 13 fatigued based on FSS (i.e., fatigued: FSS ≥ 4 and MFIS ≥ 34) Immunomodulant/immunosuppressive drugs: some patients receiving treatment (without further information) 13 HC	Central parameters: MEP amplitude and latency, CSP Peripheral parameters: femoral nerve electrical stimulation Measured at baseline and every 3 min throughout cycling during of a step test until reaching volitional exhaustion	Clinical parameters: Depression: CES-D Sleep quality: PSQI Quality of life: MSQoL-54 Perceived activity level: GLTEQ Peripheral pro-inflammatory cytokines Axial panoramic ultrasound for knee extensor cross-sectional area, actigraphy (sleep and rest-activity cycles)	Significant worse depression, sleep and quality of life scores in fatigued PwMS compared to the other groups; no group difference in actigraphy, maximal aerobic capacity and perceived activity level Higher interleukin 8 in fatigued PwMS compared to HC During cycling: No time or interaction effect was observed for MEP amplitude or latency. Reduction in CSP compared to baseline in fatigued PwMS compared to the other groups At volitional exhaustion: • Reduced MEP amplitudes and prolonged MEP latencies in fatigued PwMS; loss of group differences in CSP • Higher decline in maximal voluntary contraction force and potentiated twitch force in fatigued PwMS Regression analysis: Prolonged MEP latency, increased peripheral muscle fatigability and depression scores were significant predictors of fatigue severity

*aMT, active Motor Threshold; BDI, Beck Depression Inventory; BRPES, Borg Rating of Perceived Exertion Scale ; CES-D, Center for Epidemiologic Studies Depression Scale; CMAP, compound Motor Action Potential; CMCT, Central Motor Conduction Time; CSP, cortical silent period; DTI, Diffuse Tensor Imaging; EDSS, Expanded Disease Severity Scale; ESS, Epworth Sleepiness Scale; FSS, Fatigue Severity Scale; GLTEQ, Godin-Leisure-Time- Exercise Questionnaire; HADS, Hospital Anxiety and Depression Scale ; HDRS, Hamilton Depression Rating Scale; 9HPT, Nine Hole Peg Test; HC, Healthy Controls; IHI, Interhemispheric Inhibition; ICF, Intracortical Facilitation; MEP, Motor Evoked Potential; MFIS, Modified Fatigue Impact Scale; MSIS 29, Multiple Sclerosis Impact Scale; MSQoL-54, Multiple Sclerosis Quality of Life-54; MS, Multiple Sclerosis; PSQI, Pittsburgh Sleep Quality Index; PwMS, Patients with Multiple Sclerosis; rMT, resting Motor Threshold; SDMT, Symbol Digit Modalities Test; SICI, Short-Interval intracortical Inhibition; TAS, Toronto Alexithymia Scale; TMS, Transcranial Magnetic Stimulation; TNF, Tumor Necrosis Factor; VAS, Visual Analog Scale*.

### Treatment of Fatigue in MS Using NIBS

As stated previously, MS fatigue is perceived as a multidimensional construct, thus its management requires a personalized strategy that should address each of its dimensions. In this setting, various therapeutic interventions have been tried including pharmacological and non-pharmacological approaches. Concerning the pharmacological solutions, there is a vast array of literature on this topic, with numerous molecules being tested over the last years and only few having benefited from an in-depth evaluation. This includes amantadine hydrochloride, modafinil, pemoline, carnitine, and potassium channels blockers. Although all these drugs have demonstrated promising results in some studies, other works have failed to document any amelioration of fatigue and have thus questioned their place in the management of this symptom. Moreover, in a recent randomized, placebo-controlled, double-blind trial that compared the effects of amantadine, modafinil, and methylphenidate on MS fatigue, the studied drugs were not significantly superior to placebo in terms of efficacy and engendered more frequent adverse effects (52). Description of the mechanisms of action of these drugs and results of the corresponding studies falls outside the scope of this review [for more details, please refer to (1)].

In what concerns non-pharmacological alternatives, numerous therapies have been assessed so far and have led to some encouraging results, as has been demonstrated with exercise, whole body cryostimulation (53), cognitive behavioral therapies (CBT) (54), and NIBS (55). As mentioned in the introduction, in this review, we will only focus on the latter techniques (i.e., NIBS), the remaining does not match the main purpose of the current review.

As stated previously, tDCS is a NIBS technique that relies on the administration of a feeble electric current through two saline-soaked sponge electrodes, an anode and a cathode, placed on the scalp and connected to a battery-driven stimulator (28). The choice of the electrodes' place and polarity depends on the intended effects. This approach has been shown to be beneficial in several neurological and psychological problems, such as neuropathic pain, anxiety, and depression, to set a few. Therefore, its application in PwMS, and particularly in the context of fatigue, has been the focus of several research teams. The majority of studies that assessed the effects of tDCS on subjective or perceived self-reported MS fatigue adopted a crossover randomized (or pseudorandomized) design, were double-blinded and sham-controlled, and consisted of applying an anodal stimulation over the left dorsolateral prefrontal cortex (DLPFC), the right posterior parietal cortex, the bilateral sensorimotor cortex, the bilateral motor, or the bilateral primary somatosensory cortex. The current used was of weak intensity, ranging from 1.5 to 2 mA; and the session duration varied between 15 and 20 min. While results from bilateral somatosensory cortex/bilateral motor cortex stimulation were encouraging (56–59); those of left DLPFC were controversial, with two studies showing negative results (60, 61) and two others documenting positive outcomes (62, 63). Such a discrepancy seems to be due to the difference in the current intensity [current intensity: 1.5 mA in (60) vs. 2 mA in (62, 63)] and the number of stimulation sessions [3 in (61) and 5 in (62, 63)] across the abovementioned studies. This point of view could be supported by the data of a recent work where robust anti-fatigue effects were seen after the left DLPFC and left M1 stimulation, with more lasting fatigue reduction observed following the former condition (64).

As for the posterior parietal cortex (62) and the bilateral sensorimotor cortex (of hand area) (57), results should be interpreted with caution since they are based on two studies only, and further investigations are needed before drawing any formal conclusion.

Regarding fatigability, cognitive and motor fatigability have been investigated in three studies, two of them tested the impact of one anodal session [over the right parietal cortex in (65) and over the left DLPFC in (66)] on cognitive performance during a particular task [visual task in (65), and measurement of P300 in (66)] and one work assessed the effects of 5 consecutive anodal sessions over M1 on a cluster of symptoms including pain, subjective fatigue, and motor fatigability (67). It has been shown that delivering anodal stimulation over the left DLPFC or the right parietal cortex could counteract cognitive fatigability and prevent decrement in cognitive performance (reflected by prolonged reaction time). On the other hand, anodal stimulation of M1 would result in a decrease in motor fatigability (of the contralateral leg), as well as an amelioration of subjective fatigue and pain.

All the previously reported studies have addressed the short-lasting effects of tDCS and its feasibility over a short period of time (sessions were performed over 1 or 2 weeks). However, to suggest this innovative technique as a therapeutic solution for PwMS, we need to maintain its effectiveness over time; such maintenance requires repetition of the sessions, and this has been addressed in some case studies where sessions (14–19 sessions) were repeated over 4 weeks and ensured a long-term reduction of fatigue and amelioration of cognitive functions as well as the mood state (68, 69).

Although the results of these trials are interesting, a limitation should be considered. In fact, health providers are dealing with a fragile population, thus suggesting to this population that recurrent traveling to the care facilities is a real challenge. Often, these patients are either disabled and/or have a busy personal or professional schedule, which should be taken into consideration. Hence, the best solution would be by organizing a home-based therapy. The feasibility and efficacy of the latter have been tested by Charvet et al., and it has been documented that remotely supervised tDCS sessions are safe, could be coupled with computer-based cognitive training programs, and would help in alleviating fatigue and improving cognitive performance (70).

Besides tDCS, other neuromodulation approaches have been also tried in the setting of MS fatigue. However, the literature is limited to few studies. Two of them have explored the potential role of transcranial random noise stimulation (tRNS) in the treatment of fatigue and three of them have evaluated the place of rTMS or TBS in this context.

Transcranial random noise stimulation yielded beneficial antifatigue effects in one study (71) but not in the other one (72). Compared to Palm and colleagues, Salemi and colleagues had a different study design (crossover vs. parallel arms, respectively), applied a larger number of sessions (3 vs. 10, respectively), and targeted a different cortical site (left DLPFC vs. M1 of the dominant side or contralateral to the most affected limb, respectively).

Transcranial random noise stimulation/theta burst stimulation studies targeted different cortical sites and were applied alone or in combination with exercise or physical therapy. Some of these studies suggested promising findings that are worth replicating in future trials (73–76). Briefly, with regards to rTMS, 10–18 sessions applied at 5–20 Hz over M1 bilaterally, with or without physical therapy, resulted in significant fatigue reduction (74, 75). As for TBS, the existing literature on the matter consisted of iTBS protocols. Ten sessions of such intervention, combined with exercise or physical therapy, did not significantly affect fatigue when applied over the cerebellum or M1 bilaterally (75, 76) but yielded antifatigue effects when applied over M1 contralateral to the most spastic limb (73). The latter protocol applied without concomitant exercise did not significantly reduce fatigue compared to the sham (73). Here, it is worth stating that the considered iTBS studies primarily focused on MS spasticity, fatigue being included as a secondary outcome. Therefore, the effects of iTBS on primary MS fatigue merit to be further addressed. Details of NIBS application in MS fatigue are presented in Table 2.

**Table 2:** Summary of NIBS studies in MS fatigue.

	**Participants**	**Inclusion criteria**	**Design**	**Randomization**	**Washout interval**	**Number of stimulation sessions**	**Stimulation site**	tDCS/tRNS electrodes*or rTMS/iTBS coil position	**Stimulation parameters and session duration**	**Fatigue measures**	**Results**
**tDCS studies**											
Ferrucci et al. (59)	25 *(22 RR, 3 SP)* Immunomodulant/immunosuppressive drugs: patients receiving treatment continued taking them during the study (without further information)	MFIS > 45 EDSS <6.5	Crossover double-blind, sham-controlled	Yes	1 month	5 consecutive daily sessions	Bilateral M1	Anode: C3 and C4 Cathode right deltoid	1.5 mA and 20 min	FIS	Significant fatigue reduction up to 3 weeks after the last active stimulation session
Saiote et al. (60)	25 *(RR)* Immunomodulant/immunosuppressive drugs: 10 patients receiving treatment	FSS ≥ 4 EDSS ≤ 6	Crossover, double-blind, sham controlled	Pseudo randomization	2 weeks	5 consecutive daily sessions	Left DLPFC	Anode: F3 Cathode: contralateral forehead	1.5 mA and 20 min	MFIS, FSS, MS-SF	Absence of fatigue improvement
Tecchio et al. (56)	10 *(7 RR, 1 SP, 2 PP)* Immunomodulant/immunosuppressive drugs: Information NP	MFIS > 38 EDSS ≤ 3.5	Crossover, double-blind, sham controlled	Yes	Please refer to #	5 consecutive daily sessions	Bilateral whole body S1	Anode: personalized Cathode: Oz	1.5 mA and 15 min	MFIS	Significant decrease in fatigue scores up to 2 months following active condition. [The effects lasted up to 9.6+/- 3.6 weeks after the active condition (vs. 4.8+/- 1.8 weeks following sham condition)]
Tecchio et al. (57)	21 *(RR)* Immunomodulant/immunosuppressive drugs: Information NP	Physical subscore of MFIS > 15 EDSS ≤ 3	Crossover, double-blind, sham-controlled	Yes	Please refer to #	5 consecutive daily sessions	Bilateral whole body S1 vs. bilateral hand SM area	Anode: personalized Cathode: Oz (S1 condition) vs. under the chin (SM condition)	1.5 mA and 15 min	MFIS	Significant decrease in fatigue scores following active S1 condition (no changes after SM condition)
Hanken et al. (65)	Study 2: 46 *(18 RR, 28 SP)* Immunomodulant/immunosuppressive drugs: 67% receiving the treatment	NP	Parallel groups, double-blind, sham-controlled	Yes	NA	1 session before the performance of a visual vigilance task	Right parietal cortex	Anode: P4 Cathode: left forehead	1.5 mA and 20 min	RT on a visual vigilance task	Anodal right parietal stimulation counteracts the vigilance decrement. This effect was only observed in the setting of mild to moderate cognitive fatigue (and not in case of severe cognitive fatigue)
Ayache et al. (68)	16 *(11 RR, 4 SP, 1 PP)* Immunomodulant/immunosuppressive drugs: 13 patients receiving treatments	VAS (pain) > 4	Crossover, double-blind, sham controlled	Yes	3 weeks	3 consecutive daily sessions	Left DLPFC	Anode: F3 Cathode: AF8	2 mA and 20 min	MFIS	No effects on fatigue (It is important to mention that fatigue was assessed as a secondary outcome)
Chalah et al. (62)	10 *(8 RR, 1 SP, 1 PP)* Immunomodulant/immunosuppressive drugs: 10 patients receiving treatments	FSS > 5 EDSS ≤ 6.5	Crossover, double-blind, sham controlled	Yes	3 weeks	5 consecutive daily sessions	Left DLPFC vs. Right PPC	Anode: F3 Cathode: AF8 vs. Anode: P4 Cathode: Cz	2 mA and 20 min	MFIS, FIS and VAS	Significant fatigue reduction was obtained after left prefrontal cortex anodal stimulation but not after right parietal stimulation. Long-term effects were not assessed
Charvet et al. (70)	Study 1: 35 (20% RR in active arm, 75% RR in control arm) Study 2: 27 (40% RR in active arm, 58% RR in sham arm) Immunomodulant/immunosuppressive drugs: information NP	SDMT (z score) ≥−3 EDSS?6.5	Study 1: open label Study 2: parallel groups, double-blind, sham-controlled	Study 1: no Study 2: yes	NA	Study 1: 10 sessions^§^ Study 2: 20 sessions^§^ (Remotely supervised sessions, administered at home daily, 5 days per week)	Left DLPFC	Anode: left prefrontal cortex Cathode: right prefrontal cortex (Exact position not precised)	Study 1: 1.5 mA and 20 min Study 2: 2 mA and 20 min (Intensity was set at 1.5 mA if 2 mA was not tolerated)	PROMIS FSS VAS	Study 1: no effect on fatigue Study 2: significant fatigue reduction which was more evident in patients with higher fatigue scores
Fiene et al. (66)	15 *(14 RR, 1 SP)* Immunomodulant/immunosuppressive drugs: All patients receiving treatments	WEIMuS ≥ 9	Crossover, single blind, sham controlled	Yes	1 week	1 session	Left DLPFC	Anode: F3 Cathode: right shoulder	1.5 mA and 27–28 min	VAS Simple RT P300 components (latency & amplitude)	Active stimulation session counteracted cognitive fatigue and prevented any decrease in task performance (reflected by an increase in P300 amplitude and a stabilization of the RT)
Cancelli et al. (59)	10 *(types NP)* Immunomodulant/immunosuppressive drugs: information NP	MFIS >35 EDSS ?2	Crossover, double-blind, Sham-controlled, study	Yes	Please refer to #	5 consecutive daily sessions	Bilateral whole body S1	Anode: personalized Cathode: Oz	1.5 mA and 15 min	MFIS	Significant fatigue reduction following active stimulation.
Chalah et al. (63)	11 *(10 RR, 1 SP)* Immunomodulant/immunosuppressive drugs: 9 patients receiving treatments	FSS > 5 EDSS <6.5	Crossover, double blind, sham-controlled study	Yes	3 weeks	5 consecutive daily sessions	Left DLPFC	Anode: F3 Cathode: F4	2 mA and 20 min	FSS and MFIS	Significant fatigue reduction (i.e., a decrease of MFIS scores) that persisted up to 1 week following the last active stimulation session
Mortezanejad et al. (64)	32 *(types NP)* Immunomodulant/immunosuppressive drugs: information NP	FSS > 5 EDSS <4	Parallel groups, double blind, sham controlled	Pseudo randomized	NA	6 sessions (3 sessions per week over two consecutive weeks, sessions were administered every other day)	Left DLPFC vs. Left M1	For left DLPFC stimulation, anode over F3 and cathode over the contralateral supraorbital area For the left primary cortex, anode over C3 and cathode over C4	1.5 mA and 20 min	FSS	Significant fatigue reduction after active left DLPFC and after left M1 conditions. Only left DLPFC anodal stimulation led to long-lasting effects (up to 4 weeks following the last stimulation session)
Workman et al. (67)	6 (RR) Immunomodulant/immunosuppressive drugs: information NP	NA	Crossover, double blind, sham-controlled study	Yes	NP	5 daily consecutive sessions	Left M1	Anode: M1 representation of the more-affected leg Cathode: contralateral supraorbit	2 mA and 20 min	MFIS Motor task VAS (pain)	Improvement of fatigability, reduction of fatigue, and amelioration of pain
**tRNS studies**											
Palm et al. (72)	16 *(11 RR, 4 SP, 1 PP)* Immunomodulant/immunosuppressive drugs: 13 patients receiving treatments	VAS (pain) > 4	Crossover, double blind, sham-controlled study	Yes	3 weeks	3 consecutive daily sessions	Left DLPFC	Anode: F3 Cathode: AF8	2 mA, random frequencies range 0–500 Hz and 20 min	MFIS	No effects on fatigue (It is important to mention that fatigue was assessed as a secondary outcome)
Salemi et al. (71)	17 (RR) Immunomodulant/immunosuppressive drugs: 13 patients receiving treatments	MFIS >20 EDSS ≤ 4.5	Parallel, single-blind, sham-controlled study	Yes	NA	10 sessions (5 consecutive daily sessions per week over 2 consecutive weeks)	M1 of the dominant side or contralateral to the most affected limb	C3 + FP2 or C4 + FP1	1.5 mA, random frequencies range 100–640 Hz and 15 min	MFIS	Significant fatigue reduction after the last session
**rTMS studies**											
Gaede et al. (74)	28 (26 RR, 2 SP)	FSS ≥ 4 EDSS between 0 and 6	Parallel, semi-blind, sham-controlled study	Yes	NA	18 sessions (3 sessions per week over 6 weeks)	Left prefrontal cortex or bilateral M1	Left prefrontal cortex: H coil 5 cm anterior to the left motor hot spot parallel to the sagittal suture M1: center of the H coil-over M1	Left prefrontal cortex: 120% rMT, 18 Hz, 50 trains (train duration 2 s, ITI 20 s), 1,800 stimuli, 18 min M1: 90% rMT, 5 Hz, 40 trains, bursts of 20 stimuli, ITI 20 s, 800 stimuli, 16 min	FSS	Significant fatigue reduction mostly following M1 stimulation that lasted over 6 weeks
Korzhova et al. (75)	34 (SP) Immunomodulant/immunosuppressive drugs: None	Modified Ashworth Scale ≥ 2 at the knee joint	Parallel, double-blind, sham-controlled study Concomitant physical therapy	Yes	NA	10 sessions (5 consecutive daily sessions per week over 2 consecutive weeks)	Bilateral M1	Figure of eight coils positioned using neuronavigation over bilateral M1	rTMS: 80% rMT, 20 Hz, stimulation 2 s and ITI 28 s, 1,600 stimuli, 30 min	MFIS	Significant fatigue reduction
Mori et al. (73)	30 (RR) Immunomodulant/immunosuppressive drugs: information NP (not modified 2 months prior and during the study)	EDSS between 2 and 6 Presence of lower limb spasticity	Parallel, double blind, sham-controlled study: • iTBS alone • iTBS + exercise therapy • Sham stimulation + exercise therapy	Yes	NA	10 sessions (5 consecutive daily sessions per week over 2 consecutive weeks)	M1 leg area contralateral to the affected limb	Figure of 8 coils positioned over the optimal site evoking MEP on the contralateral soleus muscle vs. 1 cm ahead and 1 cm lateral to CZ if no detectable MEP at any leg	80% aMT, 5 Hz, 10 bursts, three stimuli per burst at 50 Hz, repeated at 5 Hz, 600 stimuli, 200 s	FSS	Significant fatigue improvement following iTBS combined with exercise therapy, but not following iTBS alone (It is worth noting that fatigue was a secondary outcome)
Korzhova et al. (75)	34 (SP) Immunomodulant/immunosuppressive drugs: None	Modified Ashworth Scale ≥ 2 at the knee joint	Parallel, double-blind, sham-controlled study Concomitant physical therapy	Yes	NA	10 sessions (5 consecutive daily sessions per week over 2 consecutive weeks)	Bilateral M1	Figure of eight coils positioned using neuronavigation over bilateral M1	iTBS: 80% rMT, 5 Hz, 10 bursts, three stimuli per burst at 35 Hz, repeated at 5 Hz, 1,200 stimuli, 10 min	MFIS	No changes in fatigue
Tramontano et al. (76)	16 (9 SP, 7 progressive relapsing) Immunomodulant/immunosuppressive drugs: information NP	EDSS between 4.5 and 6.5 Modified Ashworth scale ≤ 1 at the leg	Parallel, double-blind, sham-controlled study Concomitant exercise-based vestibular rehabilitation	Yes	NA	10 sessions (5 consecutive daily sessions per week over 2 consecutive weeks)	Bilateral cerebellum	Figure of eight coils over the left and right cerebellum	Two runs of iTBS over both the right and left cerebellum separated by a 5 min interval	FSS	Significant fatigue reduction following iTBS (It is worth noting that fatigue was a secondary outcome)

*aMT, active Motor Threshold; DLPFC, Dorsolateral Prefrontal Cortex; EDSS, Expanded Disability Status Scale; iTBS, intermittent Theta Burst Stimulation; ITI, Intertrain Interval; FIS, Fatigue Impact Scale; FSS, Fatigue Severity Scale; M1, Primary Motor Cortex; MEP, Motor Evoked Potentials; MFIS, Modified Fatigue Impact Scale; MS-SF, Multiple Sclerosis-Specific Fatigue Scale; NA, Not Applicable; NP, Not Provided; PP, Primary Progressive; PPC, Posterior Parietal Cortex; PROMIS, Patient-Reported Outcomes Measurement Information System; rMT, resting Motor Threshold; RR, Relapsing Remitting; RT, Reaction Time; rTMS, repetitive Transcranial Magnetic Stimulation; S1, Primary Somatosensory Cortex; SM, Sensorimotor; SP, Secondary Progressive; tDCS, transcranial Direct Current Stimulation; VAS, Visual Analog Scale. WEIMuS, Würzburger Fatigue Inventory for MS*.

*Electrodes position is defined according to 10−20 EEG international system.

*#Washout is considered completed when half of the tDCS effect is lost (i.e., in fact, MFIS was obtained each week following the last session of each block, when the MFIS increment met the criteria of the following formula: $\frac{MFIS\;\left(washout\;time\right)-\;MFIS\left(before\;fisrt\;session\right)}{MFIS\;\left(before\;fisrt\;session\right)}<0.5\;\left(\frac{MFIS\;\left(after\;the\;last\;session\right)-\;MFIS\left(before\;fisrt\;session\right)}{MFIS\;\left(after\;the\;last\;session\right)}\right)$; it reflected the end of the washout period, the second block could then be administered)*.

*§Sessions were combined with a computer-based cognitive training program*.

*Case reports are not included in this table*.

## Conclusion

This review explored the potential role of neurophysiology in the exploration and modulation of fatigue in PwMS. First, in terms of pathophysiology, the available studies that included intracortical excitability and corticospinal excitability outcomes yielded inconsistent findings. For instance, while fatigue was correlated with SICI/CSP (GABA-mediated outcomes) in some studies, such a correlation was not found in other studies. The included studies were cross-sectional; they assessed fatigue using different scales and included PwMS suffering from different disease subtypes. This highlights the relevance of longitudinally studying the dynamics of these parameters across the disease course and subtypes and their relationships with fatigue. In addition, considering secondary factors to fatigue and taking into consideration the symptom cluster in the covariate analysis would also be of help (3). Besides tackling the previously mentioned differences (subjected or perceived self-reported fatigue vs. fatigability, primary vs. secondary), the temporal dimension of fatigue merits to be considered. In this perspective, Palotai and colleagues longitudinally assessed PwMS and suggested different types of fatigue (sustained fatigue vs. one time-point fatigue vs. reversible fatigue), which seem to differ in brain imaging findings (brain parenchymal fraction, T2 lesion volume) (77), a finding that might also apply to corticospinal excitability parameters.

Second, in terms of tDCS, the data altogether suggest promising tDCS effects obtained on MS fatigue. The current challenge remains to find the best parameters to optimize treatment effects (e.g., applying a higher number of sessions, selecting the best cortical target, selecting the best return electrode location, designing patient-tailored electrodes, increasing the current intensity up to 4 mA) (56, 57, 68, 69, 78). As stated with neurophysiological exploration, it would be helpful to consider the temporal dynamics of fatigue and the symptom cluster when assessing the mediators of response to tDCS. It is noteworthy that, when it comes to either exploring or modulating MS fatigue using NIBS techniques, a confounder that needs to be considered or accounted for is the pharmacological profile of the recruited cohorts. For instance, some medications (e.g., disease-modifying therapies, symptomatic treatments) might modify the corticospinal excitability in PwMS (79). In addition, some treatments (e.g., sodium channel blockers, calcium channel blockers, medications that act on neurotransmitters pathways) may also affect the tDCS effects on corticospinal excitability (80). The relationship between the treatment status and the considered outcomes (e.g., SICI, ICF, IHI, CSP, or fatigue improvement) warrants further investigation since this was rarely or not tackled in previous studies.

Third, studying the effects of tDCS on corticospinal excitability would provide further insights into the neurophysiological mechanisms of fatigue and the antifatigue mechanisms of action of tDCS (57, 68).

Finally, home-based tDCS will provide a solution for physically disabled PwMS. The application of psychotherapies (e.g., CBT-based online interventions) and pharmacotherapy might yield synergistic effects (81). Such an approach constitutes a domain that remains to be explored in this context.

## Author Contributions

SA and MC: conceptualization and methodology. MC, NS, GA, and SA: data analysis, writing—original draft preparation, and writing—review and editing. SA: supervision. All authors have read and agreed to the published version of the manuscript.

## Conflict of Interest

SA declares having received compensation from ExoNeural Network AB, Sweden. MC declares having received compensation from Janssen Global Services LLC, ExoNeural Network AB, Sweden, and Ottobock, France.

The remaining authors declare that the research was conducted in the absence of any commercial or financial relationships that could be construed as a potential conflict of interest.

## Publisher's Note

All claims expressed in this article are solely those of the authors and do not necessarily represent those of their affiliated organizations, or those of the publisher, the editors and the reviewers. Any product that may be evaluated in this article, or claim that may be made by its manufacturer, is not guaranteed or endorsed by the publisher.
